# Iowa Gambling Task and Distortion in Perception of Body Image Among Adolescent Women With Eating Disorders

**DOI:** 10.3389/fpsyg.2020.02223

**Published:** 2020-08-31

**Authors:** Concha Martínez-García, Cecilio Parra-Martínez, Ángel T. Parra, Tomás E. Martínez-García, Jose-Ramón Alameda-Bailén

**Affiliations:** ^1^Department of Developmental and Educational Psychology, University of Huelva, Huelva, Spain; ^2^Department of Chemistry, Faculty of Experimental Sciences, Research Center for Natural Resources, Health and the Environment (RENSMA), University of Huelva, Huelva, Spain; ^3^Department of Medicine, University of Seville, Seville, Spain; ^4^Department of Internal Medicine, Juan Ramón Jiménez Hospital, Huelva, Spain; ^5^Department of Clinical and Experimental Psychology, University of Huelva, Huelva, Spain

**Keywords:** adolescent women, decision making, distortion of body image, eating disorders, Iowa gambling task, perception

## Abstract

The Iowa gambling task (IGT) is an instrument for the neuropsychological evaluation of cognitive and emotional decision making (DM) processes that was created to test the somatic marker hypothesis (SMH) described by Damasio in 1994. It was initially applied to patients with frontal lobe lesions due to its association with executive functions but was subsequently used on patients with a variety of disorders. Although the DM process is inherently perceptual, few studies have applied the IGT to examine DM processes in patients with eating disorders (EDs), and even fewer have associated the IGT to the perceptual distortion of body image (PDBI) in this population. People diagnosed with ED exhibit heightened control over their somatic responses—for example, they can delay digestion for hours—and DM may be affected in this condition. This study compares the performance of two samples of adolescent women—hospitalized patients with ED, and healthy controls with similar demographic characteristics—on the IGT using body image as a possible factor in the SMH. Seventy-four women with a mean age of 14.97 years (SD = 2.347) participated. To analyze their body self-image, we used the figure-rating scale and compared the results with their body mass index (BMI). Correlations between indices of the IGT and distortion in body image were then explored. The results revealed significant differences between the groups in terms of evolving performance on the partial IGT. Patients with ED performed worse than their healthy counterparts in the last 40 trials and exhibited greater distortions in their body image, especially in terms of overestimation. Indices of these distortions were negatively correlated with the total IGT. These results are compatible with the SMH because they suggest that patients with ED evinced blindness with regard to the future, as described by their authors. In addition, a negative correlation was found between the IGT and PDBI, showing that a more distorted body image was associated with lower IGT, that is, more disadvantageous or riskier decisions were made by the subjects with more distortion.

## Introduction

Decision making (DM) encompasses multiple and varied situations, ranging from the simplest choice of constant everyday decisions to the most complex situations at crucial points in our lives. In such situations, cognitive processes and neurological, somatic, and emotional structures are involved in initiating, supervising, controlling, and evaluating our behavior. These neurological systems are involved in executive functions (FFEE) and higher psychological processes. At the same time, the most basic psychological processes like attention, perception, memory-related, and motivational systems must function properly to enable complex or higher processes, such as DM (Fox et al., 2013; Patterson et al., 2014; Cowan, 2017; Padilla et al., 2018). Neuropsychological functioning enables human beings to adapt to their environment, to assume diverse responsibilities and tasks, display socially appropriate behavioral and emotional responses, overcome and learn from adversity, and formulate abstract thoughts of self-awareness and ethical and moral judgments (Pelegrín and Tirapu, 1995; Graham and Harris, 1996; Stelzer et al., 2010).

Recent neuroscientific studies have shown that emotions are crucial in the DM process. Decisions and their consequences imply emotions, and many of our choices are guided by past emotional experiences or their anticipation. This evidence has led to the hypothesis that emotions can play an important role in guiding our decisions, as described in the somatic marker hypothesis (SMH) proposed by Damasio (1994). To test this, the Iowa gambling task (IGT) (Bechara et al., 1994, 1997) was created. It was used initially on patients with frontal lobe damage and was subsequently administered to people with a wide variety of psychopathologies.

The IGT is designed to require more learning than is needed to simply indicate correct and incorrect long-term responses because it allows subjects to fine-tune their performance depending on feedback in the form of the consequences of their responses in prior trials (Pasion et al., 2017). Kovalchik and Allman (2006) used the IGT to study reverse learning conceptualized as the shift toward a more adaptive response (less attractive because of reduced gains) by inhibiting the prepotent response (more attractive because of large initial gains). This adaptive behavior of inhibiting the predominant response requires that participants learn to forgo high monetary rewards in favor of low to moderate monetary rewards. Likewise, non-inhibition would be comparable to the characteristic behavioral deterioration observed in patients with ventromedial prefrontal cortex (VMPFC) damage, addicts, psychopaths, and individuals with other self-destructive disorders who persist in previously gratifying behavior despite high long-term costs.

Therefore, the learning that can indicate the IGT is not the simple change from incorrect to correct answers but rather requires active inhibition of the instinctive response to choose the less attractive option.

Perception is fundamentally a psychological process that does not imply a copy of empirical reality but the interpretation made by the subject in an active and constructive way using content captured by the senses (Mohr et al., 2016; Felin et al., 2017). This interpretation is based on past perceptual experiences, their consequences, and personal expectations and predispositions (Biggs et al., 2015; Soto et al., 2015).

Adolescence is the stage of development in which one’s identity, self-concept, and self-esteem are acquired, with the perceived body image as the main source of feedback. The body image is evolutionarily constructed by internalizing the experiences of his/her own body through interpersonal, social, and cultural mores that dictate personal attractiveness and competence (Rodgers et al., 2014; Rohde et al., 2015; Haynos et al., 2016). Therefore, it is not unusual that during this stage of higher vulnerability to sociocultural influence and peer group influence, teenagers assign a greater weight to their self-evaluation of their physical image than to other maturation-related factors of development, such as DM and its consequences, even though they also shape the emerging adult personality (Brosch et al., 2013; Hartley and Somerville, 2015).

### Age and Performance in the IGT

IGT performance has been shown to increase significantly from early adolescence to adulthood. Hooper et al. (2004) compared IGT performance in groups of children and adolescents of various ages, obtaining significantly better results with older subjects. Participants ages 14–17 performed significantly better than participants ages 9–10 in the 100 computerized IGT trials. This was also shown with the 5-block analysis (20 cards/block). In Block 4, the 14- to 17-year-old group performed better than the two younger groups (9–10 and 11–13 years old). In Block 5, the 14- to 17-year-old group performed better than the 9- to 10-year-old group. Overman and Pierce (2013) confirmed these results but also observed that once subjects reached young adulthood (mean age of 19 years), they no longer differed in performance compared to older adults (mean age of 59 years).

This steady increase in IGT performance from adolescence through young adulthood has been interpreted in different ways, one of which is that increases in performance are related to ongoing neuroanatomical and neurochemical development of the frontal lobe.

During adolescence there are substantial brain changes, especially in the PFC (Giedd et al., 1999), making IGT an optimal task to use with this population. The hypotheses that attribute poor decision making among adolescents to neuroanatomical changes in areas within the PFC indicate that optimal IGT performance depends on the integrity of several PFC regions: the orbital PFC (ORBPFC) (Bechara et al., 1994), dorsolateral PFC (Fellows and Farah, 2005), and/or dorsomedial PFC (Manes et al., 2002). Damage to any of these areas impairs IGT performance and, in adolescence, these and other areas of the brain undergo changes.

### Gender and IGT Performance

Recent studies on gender differences in IGT performance show that males perform better than females. This gender difference is the result of women’s preference for high-gain-frequency cards, either from the disadvantageous Deck B (van den Bos et al., 2013) or from the summation of the two decks, B and D (Reavis and Overman, 2001; Overman et al., 2006). In addition, men declared the correct rule significantly earlier on the task (75th test on average) than women (97th test on average). That is, men and women learn to choose advantageous cards in the task, but men choose more cards from decks C and D and women choose more cards from decks B and D.

Different etiological hypotheses are proposed for these results. Some authors proposed that hypersensitivity to loss may be driving female performance in risk-taking tasks (Deakin et al., 2004; Lee et al., 2009; van den Bos et al., 2012, 2013), while other authors have interpreted that the IGT gender difference is driven by a male aversion to loss and a female preference for reward (Overman et al., 2006; Overman and Pierce, 2013). This second hypothesis is based on the results of Bolla et al. (2004), who reported differential activation in ORBPFC subregions, measured with PET imaging, between men and women while performing the IGT. Men showed increases in activation in the lateral ORBPFC (BA 47), and females showed increases in activation in the medial ORBPFC (BA 11).

Regarding the hypothesis of hypersensitivity to loss in women, van den Bos et al. (2013) reported that they focus on both win-loss frequencies and long-term pay-off of decks, while men focus on long-term pay-off. In addition, women may be more sensitive to occasional losses in long-term advantageous decks than men. Therefore, women need more trials (40–60) to reach the same performance level as men.

This proposal is based on the psychological mechanisms and neurobiological substrates underlying sex differences in IGT-type decision-making: serotoninergic activity and left-right hemispheric activity, as well as differences in the dorsolateral prefrontal cortex. Sex differences in ORBPFC activity may be due to the organizational effects of gonadal hormones. Thus, the behavioral and neurobiological differences in IGT between men and women would be an expression of more general sexual differences in emotional regulation.

ORBPFC involvement in IGT performance leads to the promptly assumption that enhanced IGT performance is based on functional maturation of ORBPFC and related networks. However, it should not be forgotten that there are numerous social and environmental changes that occur concomitantly with IGT improvement during this time period (He et al., 2012; Overman and Pierce, 2013).

### Perceptual Distortion in Adolescent Women With Eating Disorders (ED)

Alterations of cognitive processes involved in DM, such as perception, have a direct impact on a person’s ability to develop independent, autonomous, and adaptive personal and social lives (Rolls, 2004; Brooks et al., 2016). Thus, distortions in perception, particularly those related to body image, are considered to be among the main clinical symptoms of eating disorders (EDs). Alterations in perception have been observed in other psychological disorders, such as body dysmorphic disorder, hypochondria, and psychosomatic delusions, and have been noted in the general or non-clinical population. Perceptual distortion of body image (PDBI) is also common in adolescents of control groups). The literature suggests that ∼60% of adolescent females are dissatisfied with their bodies, and desire to change their shapes or sizes (Balluck et al., 2016; Coelho et al., 2016; Duchesne et al., 2017; Japil et al., 2018; Quittkat et al., 2019). It also calls attention to the increasing trends in these factors across the world (Smink et al., 2012; Mond et al., 2013; Ganesan et al., 2018; Fuller-Tyszkiewicz, 2019).

Research on sociocultural influences on body image development has had extensive media impacts (Rodgers et al., 2014). However, little attention has been paid to describing the basic psychophysiology of body image and analyzing its components in the context of personal health and well-being. It is also important to consider the aesthetic industry, with an objective to make money from people who are unhappy with their bodies (American Society of Plastic Surgeons [ASPS], 2018; British Association of Aesthetic Plastic Surgeons, 2014; Department of Health (UK), 2013; Grogan, 2017).

In 2009, the International Society of Aesthetic Plastic Surgery (International Society of Aesthetic Plastic Surgery [ISAPS], 2009) published its first survey of interventions in plastic and cosmetic surgery. A survey of registered interventions in 20 countries, featuring 20,000 plastic surgeons of the 32,000 registered in these regions, yielded a total of 17,295,557 interventions. The most common surgery performed was liposuction, accounting for 18.9% of the total (3,268,860.273), at an average cost of $6000 US/intervention, with a total annual cost of $19,613,161,638. In 2018, the largest increases in surgical procedures was recorded in liposuction and abdominoplasty, and cases of each increased by >9.7% compared with 2017 statistics (available at: http://www.isaps.org). It is also necessary to consider these figures in light of interventions that were performed but not registered on official accounts, non-surgical treatments (medication and body creams for fat absorption, herbal treatment, slimming clothes, and cosmetics), and records that were inaccessible due to doctor–patient confidentiality agreements.

In general, the above trend aims at a social and cultural model encouraging the “beauty of slimness” or a “thin-ideal” as well as “weight phobia.” This has pernicious consequences for health and leads to an increase in eating disorders, and has led to a collective obsession with body image (Smith et al., 2018; Grave et al., 2019). Furthermore, Suisman et al. (2012, 2014) concluded that most variance in the internalization of the thin ideal can be accounted for by environmental factors, and twin models showed no significant differences in etiological effects across development, suggesting that the thin ideal is independent of genetic influences.

Neurologically, functional magnetic resonance imaging now allows us to identify areas of the brain that are over- or under-active in patients when they are exposed to controlled stimuli, such as those related to EDs. Diffusion tensor imaging allows for the dynamic mapping of circuits that connect key areas involved in executive functions: The ventromedial prefrontal cortex (VMPFC) is critical for weighing risks and rewards, learning from experience, and emotion regulation. The dorsolateral (DLPFC) is responsible for impulse inhibition and future orientation, and is connected to the anterior cingulate cortex (ACC, surrounding the corpus callosum), which is in turn connected to limbic system regions that handle reward and task anticipation, attention, detection of errors, DM, empathy, emotional modulation, and the autonomic control of pulse and blood pressure (Starr and Kreipe, 2014).

However, the frontal cortex is heterogeneous, and its development is extremely complex because not all subareas develop simultaneously during adolescence. There is specific regional development with some areas being pruned while others show increases in synapses (Giedd et al., 1999). Some researchers have suggested that changes in the dorsolateral PFC are more correlated with adolescent decision-making behavior patterns (Lewis, 1997; Sowell et al., 2001; Paus, 2005), while others have suggested that changes in the ventromedial PFC are more highly correlated with such patterns (Hooper et al., 2004; Schwartz et al., 2010).

The review by Chen et al. (2018) on the neurocognitive basis of ED in relation to decision-making indicates that activity in these brain systems is comparable to the literature knowledge on addictive and problematic behaviors. It proposes an integrative triadic neural model to give etiology to the components and altered neuropsychological functioning of compulsive food consumption based on three systems: (a) an impulsive system (fast, autonomous, and subconscious) of processing and anticipation of hyperfunctional reward (dependent on amygdala-striatum) in response to food-related cues; (b) a reflective (slow, deliberative, and conscious) system of reflective control and functional inhibition (dependent on the PFC) that cannot adequately anticipate and weigh future results; and (c) an altered interoceptive consciousness system (dependent on the insular cortex). That third system would be incorporated into the traditional dual vision and constitutes the basis of the triadic model, whose function is to integrate homeostatic signals. When it is violated, it translates into a strong desire to consume that hijacks the inhibitory system and excites the reward system.

The tripartite model of decision-making regarding food cues reveals that food-related stimuli can trigger habitual involuntary bottom-up desire mediated by the amygdala-striatal system. The goal-directed reflective system may fail to anticipate future results of excessive food consumption and/or failure to inhibit excessive food consumption. This imbalance may be exacerbated by an altered interoceptive awareness system that hijacks inhibition/reflection resources and excites the impulsive system (Chen et al., 2018).

A review by ter Steege and Kolkman (2012) indicated that during physical exercise, the increased activity of the sympathetic nervous system redistributes blood flow from the splanchnic organs to the working muscles. With prolonged duration and/or intensity of exercise, splanchnic blood flow may decrease by 80% or more. In this respect, and in search for the ideal of the body image, people with eating disorders usually practice intense physical exercise, exhibiting high control of their somatic responses, and, in extreme cases, can even delay their digestion for hours (Dubois et al., 1979; McCallum et al., 1985). They can also decide to vomit to lose weight as a short-term reward to achieve the ideal of the body image, neglecting long-term consequences (e.g., malnutrition, death).

The risky decisions made by adolescents with EDs may indicate that they have altered cognitive processes: in eating restrictions of anorexia nervosa (AN) with abnormal activity in the ventromedial PFC (Cavedini et al., 2004; Tchanturia et al., 2007; Danner et al., 2012; Chan et al., 2014) and in abusive and compulsive eating in obesity, with neuronal changes in the key neuropsychological systems involved in habits, decision-making and self-control processes (Madrigal et al., 2000; He et al., 2014, 2015; Suchan et al., 2015; Chen et al., 2018). The cognitive impairment underlying perceptual distortion of body image in EDs has also been extensively studied, as the most important component and criterion for the diagnosis of AN of DSM-V (American Psychiatric Association [APA], 2013). Studies have reported alterations in the perceptual process, decreased parietal cortex activity, and altered somatosensory integration (Hebebrand et al., 2004; Uher and Treasure, 2005; Steinglass et al., 2006; Cucarella et al., 2012), as well as increased activation in bilateral frontal structures including the medial frontal gyrus and left medial temporal gyrus covering the striatum (Hodzic et al., 2009). However, the relationships between the perceptual distortion of body image and the neuropsychological process of decision making have not been systematically explored, although both seem to concur alterations on these cortical areas.

The main objective of this study was to analyze the relationship between the perceptive distortion of one’s own body image and risk decision making using the IGT (Bechara et al., 1994). We compared a group of adolescents with ED (g. clinic) with a group of healthy and demographically similar women (g. control). The following secondary objectives were specified in the form of hypotheses to be tested: (a) the clinical and control groups will differ in the total IGT performance, but both will be negative due to their young age; (b) IGT learning will show differences between groups in the two last blocks; (c) PDBI will show greater overestimation of body image in the ED group compared to control; and (d) PDBI will correlate with IGT.

## Materials and Methods

### Participants

Seventy-four female volunteers with a mean age of 14.97 years (SD = 2.347) participated in this study. They were separated into two groups: an ED group, consisting of women hospitalized with EDs (*n* = 23), with an average age of 15.13 years (SD = 2.528), all ED group patients were hospitalized by anorexia nervosa except for one patient with bulimia. The control group included healthy women (*n* = 51), and the mean age was 14.9 years (SD = 2.283). We enrolled twice the number of healthy subjects as controls based on a recommendation of the ethics committee for clinical trials of the Juan Ramón Jiménez (JRJ) Hospital of Huelva (Spain). Age was not significantly different between groups [*t*_(__74__)_ = 0.385, *p* = 0.701].

All subjects in the ED group were undergoing the same medical and psychological treatment according to JRJ Hospital protocol.

The ED group inclusion criteria were hospitalization or having been cited as an out-patient for medical follow-up after recent hospital discharge. Subjects in the control group were students of a secondary education center with parental confirmation of no ED diagnosis. The exclusion criteria were: (i) drug consumption, including the intake of more than 12 g of ethanol per day; (ii) any psychiatric diagnosis (apart from ED); and (iii) any psychophysiological condition that could have altered the results of the questionnaire, including chronic illness and sensory or cognitive disability.

The participants were individually evaluated while ensuring the privacy of their data. ED patients usually ask physicians for study results, even though this is contrary to the course of clinical intervention. Thus, the evaluators did not inform the participants about the data obtained from these measures.

All participants and their legal tutors provided informed consent. This study was configured according to the protocols established by the PEIBA (Portal de Ética de la Investigación Biomédica de Andalucía). Ethical approval was obtained based on the relevant legislation and the national and institutional guidelines, and was in accordance with the ethical rules of the Helsinki Declaration.

### Decision Making: Iowa Gambling Task

#### Total IGT

To assess the participants’ DM, the IGT was applied using the Cartas software (Palacios et al., 2010), a computerized version of the IGT proposed by Bechara et al. (1994): the “ABCD” version. The total IGT over 100 trials of each participant was calculated as follows:

Total⁢IGT=(C+D)-(A+B)

A higher IGT score reflected better decision making while lower ones indicated riskier DM or disadvantageous choices.

The IGT consists of four decks of cards presented on a screen from which participants must choose one card in every trial for a total of 100 choices, each displaying a certain gain or loss, and the cards were distributed in four decks of 40 each. The decks were shown to the participants in the follow order: A, B, C, and D. The choice of a card was locked within 2 s of the participants clicking on it. However, participants could freely think before making a new decision without any maximum time restriction to click on the next card.

The participants started the test with €2000 in virtual currency, which was shown on the screen and automatically updated with gains or losses after each selection. The goal of each participant was to win as much money as possible. The best strategy was to choose two advantageous decks, C and D, with long-term gains, but each card in these decks had a small value (of gain or loss). On the contrary, the two disadvantageous decks, A and B, incurred long-term losses because each card had a large value (of gain or loss). A few cards offered a substantial gain (€100), but others led to large loss (-€1250).

#### Partial IGT

The partial IGT was calculated by analyzing the results of five partial blocks (20 trials per block) of the task from b1 to b5: b1 (cards 1–20), b2 (cards 21–40), b3 (cards 41– 60), b4 (cards 61–80), and b5 (81–100). The learning phase of the IGT was composed of the first three blocks, and the task execution phase was constituted by the last two blocks of the tests.

Disadvantageous decks are more attractive owing to their high rewards, and as a consequence initially attracted a prepotent response. In adaptive learning for the IGT, inhibition or curbing these initially predominant choices (decks A and B) in favor of lower monetary rewards in the short term (decks C and D) is required to obtain long-term benefits. This curbing is conceptualized as a reversal of the effect of learning (Kovalchik and Allman, 2006). During the learning phase of the IGT, implicit feedback is comparable to uncertainly experienced in real-life decisions (Bechara, 2007; Brevers et al., 2013). In its execution phase, the reversal learning effect may highlight the time at which the participants learn the advantageous strategy (Pasion et al., 2017).

### Perceptual Distortion of Body Image (PDBI)

#### Anthropometric

We first calculated the real body mass index (BMI), measuring the weight in kilograms and height in meters, using the following formula:

$$\text{BMI}=\frac{\text{weight}\,\left(\text{kg}\right)}{{\text{height}}^{2}\,\,\left({\text{m}}^{2}\right)}$$

#### Body Image

The silhouettes test (ST) was used to analyze each participant’s self-estimated body image using an adaptation of figure-rating scale proposed by Stunkard et al. (1983). In this test, graphic representations of nine rank-ordered human female figures that incrementally increased in size from underweight to obese (from F1: BMI < 18, to F9: BMI > 32) were provided to the participants. The original ST also included nine human male figures that were not used here.

The participants were instructed to select the figure perceived as representative of their own at the time of the study, and this choice was designated as the perceived BMI (pBMI). The previously measured real BMI values were classified into nine categories corresponding to the Stunkard figures as follows: F1 (BMI < 18), F2 (BMI 18–20), F3 (BMI 20–22), F4 (BMI 22–24), F5 (BMI 24–26), F6 (BMI 26–28), F7 (BMI 28–30), F8 (BMI 30–32), and F9 (BMI > 32). We refer to these results as the “current BMI” (cBMI).

#### Perceptual Body Image Distortion Index

To calculate the PDBI index, the following formula was applied:

PDBI=pBMI-cBMI

We also adjusted the PDBI as the cPDBI to include the closest upper and lower ST figures in relation to the figure selected by each participant (± 1 Fx), according to a principle of visual similarity, to reduce the possibility of false positives. For example a patient selecting F7 and having a cBMI of F4 would present a PDBI of 3 points (F7 minus F4) but a cPDBI of 2 points because we include F6 and F8 since they are the closest figures to F7, and the difference will take the figure closer to cBMI (F6, the closest figure from the range F6-F8 minus F4). In other words, we subtract 1 point to the absolute value of the PDBI except for cases with no distortion (0 points of PDBI).

The correlations between variables of the IGT and those of ST (PDBI) were calculated using Kendall’s τb non-parametric contrast test, also pBMI and cBMI were correlated. All *p* values were two-sided, with *p* < 0.05 considered statistically significant.

### Statistical Analysis

The Student’s *t*-statistic was used to compare mean values of the variables. To compare the mean socioeconomic levels of the groups, we used the non-parametric Mann–Whitney’s *U* contrast test. The Student’s *t*-test for independent samples was used to examine possible differences between groups in terms of overall DM during the test (IGT). It was also used to establish differences in anthropometric measures between groups. Repeated measurements analysis of variance (ANOVA) followed by planned comparisons of groups (ED vs. control) was used to confirm differences in the number of choices depending on the type of deck (advantageous vs. disadvantageous) and to compare group differences in terms of partial IGT (b1, b2, b3, b4, b5) to observe their evolution in performing the task across blocks. ANOVA was also used to examine the cBMI, ST, PDBI, and cPDBI. The correlations between variables of the IGT and those of ST (PDBI) were calculated using Kendall’s τb non-parametric contrast test. All *p* values were two-sided, with *p* < 0.05 considered statistically significant. Finally, we performed linear regression of the correlated variables.

## Results

### Demographic and Socioeconomic Variables

No significant differences were observed between the groups in age [*t*_(__74__)_ = 0.385 and *p* = 0.701] or socioeconomic level [*U*_(__74__)_ = 553.5; *p* = 0.658] in a questionnaire administered to parents included a scale of three options: (1) Rental housing/VPO, (2) mortgaged home, and (3) home ownership. It also asked: Is your family economy on the Andalusian per capita income average, and can you make ends meet without financial difficulties? The answer was YES or NO. Both groups were in the range of the Andalusian average per capita income.

The education levels of all participants were identical due to their age, which placed them along compulsory schooling at the secondary level (ESO) and furthermore, none of them was in a higher or lower grade than in the one corresponding to them by age.

### Decision Making

#### Total IGT: Hypothesis (a)

The normality goodness-of-fit test of the IGT variables was performed using the Kolmogorov-Smirnov statistic, and they were not significant (*p* > 0.05), indicating that the variables analyzed were normally distributed. There were no significant differences between the ED group (*M* = −4.78, SD = 15.08) and control group (*M* = −0.39, SD = 13.49) in terms of total IGT [*t*_(__74__)_ = −1.249, *p* = 0.216]. Although this index was lower in the ED group, both groups had negative values, indicating risky DM in both.

#### Partial IGT: Hypothesis (b)

**FIGURE 1:**
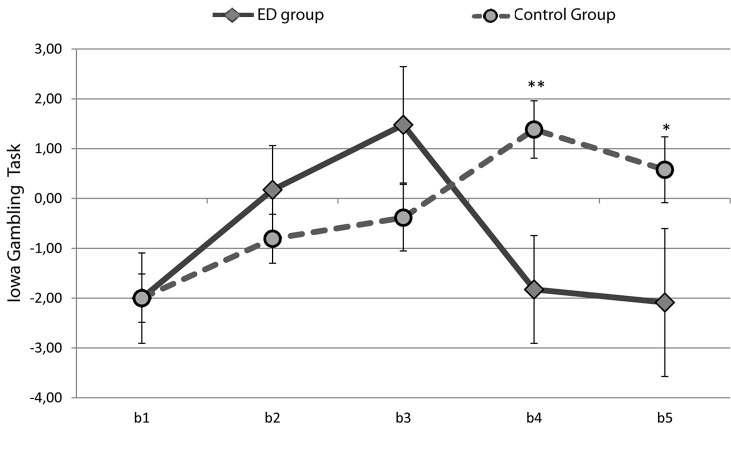
Mean IGT scores through the five trial blocks of the IGT for the ED and control groups. ^∗∗^*p* < 0.01, ^∗^*p* < 0.05.

In terms of partial IGT, we analyzed differences between the ED and control groups for each block separately, and we found an evident group effect in b4 [*F*_(__1_, _72__)_ = 8.949, *p* = 0.004] and b5 [*F*_(__1_, _72__)_ = 4.109, *p* = 0.046]. Student’s *t*-tests revealed significant differences in blocks b4 (*t* = −2.991, *p* = 0.004) and b5 (*t* = −2.027, *p* = 0.046). These differences between groups in terms of DM task evolution reflected significantly disadvantageous execution in the last 40 trials by the ED group, with more advantageous choices made by the control group (Figure 1).

These results indicate that more disadvantageous choices were made by members of both groups in the first 20 trials (first block). However, advantageous decisions increased in the second block. Only in the last two blocks of the task were significant differences observed between the groups: advantageous decisions by members of the control group increased, but such decisions decreased for the ED group, and its number of disadvantageous decisions increased. Thus, performance in different task blocks revealed differences between the ED and control groups in the last two blocks (b4, b5), without differences in the first three blocks (b1, b2, b3).

### Perceptual Distortion of Body Image: Hypothesis (c)

#### Anthropometric Measures

**FIGURE 2:**
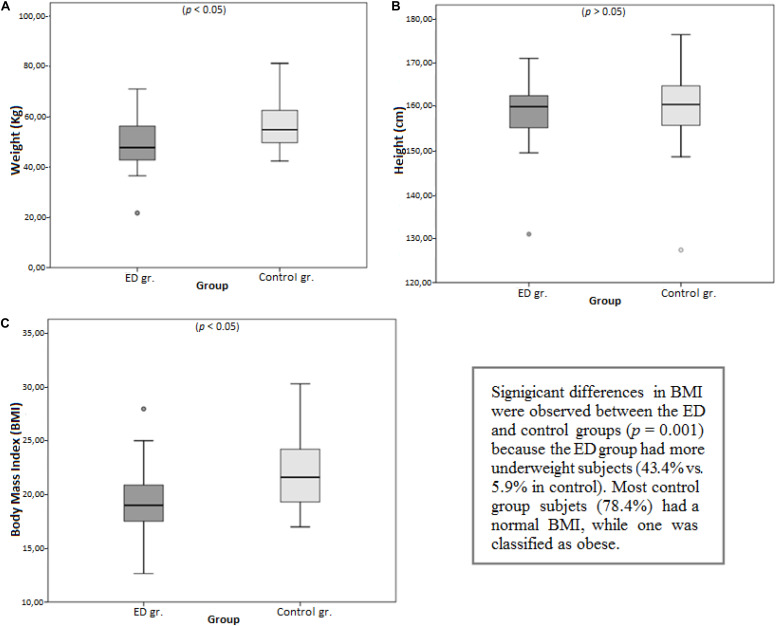
Mean comparisons between groups for **(A)** weight, **(B)** height, and **(C)** BMI.

To estimate the PDBI, we calculated the mean anthropometric indices for each group and compared them using Student’s *t*-tests (Table 1). Weight (*p* = 0.004), and BMI (*p* = 0.002) were significantly different between the control and ED groups. Height was similar between groups (*p* = 0.405), which was expected owing to the sociodemographic similarities of the samples (Figure 2).

**TABLE 1:** General anthropometric measures in the ED and control groups.

Anthropometry	Group	*N*	Mean	SD	*t*	^∗^*p*
Weight (kg)	Control group	51	56.388	8.930	−3.008	0.004
	ED group	23	49.253	10.519		
Height (cm)	Control group	51	160.010	8.001	−0.837	0.405
	ED group	23	158.330	7.968		
BMI	Control group	51	22.074	3.190	−3.171	0.002
	ED group	23	19.481	3.395		

*p values were calculated with Student’s *t*-tests.

#### Body Image

The ED and control groups did not differ in the choice of the ST figure independently of the current BMI [*F*_(__1_, _72__)_ = 0.281; *p* = 0.597] of their members. The absence of significant differences in ST between the ED and control groups contrasted with the previously reported results of the “real BMI,” which supported the hypothesis of perceptual distortion in the ED group (Table 2).

**TABLE 2:** Distribution of self-perceived figure (ST) and “current BMI” by group.

*ST Figure*		Self-perceived Figure		Current BMI	
		Control group	ED group	Control group	ED group
1	Count	0	0	2	9
	Group%	0.0%	0.0%	3.9%	39.1%
2	Count	3	3	16	4
	Group%	5.9%	13.0%	31.4%	17.4%
3	Count	7	1	9	5
	Group%	13.7%	4.3%	17.6%	21.7%
4	Count	8	7	10	3
	Group%	15.7%	30.4%	19.6%	13.0%
5	Count	8	4	8	1
	Group%	15.7%	17.4%	15.7%	4.3%
6	Count	14	2	3	1
	Group%	27.5%	8.7%	5.9%	4.3%
7	Count	9	4	2	0
	Group%	17.6%	17.4%	3.9%	0.0%
8	Count	2	2	1	0
	Group%	3.9%	8.7%	2.0%	0.0%
9	Count	0	0	0	0
	Group%	0.0%	0.0%	0.0%	0.0%

#### Perceptual Body Image Distortion Index

The ED group showed significantly higher rates of perceptual distortion of body image (PDBI) [*F*_(__1_, _72__)_ = 5.021; *p* = 0.028], particularly in the sense of overestimation (*M* = 2.52, SD = 1.675) compared to the control group (*M* = 1.59, SD = 1.651). The same calculations were made to obtain cPDBI and yielded similar results [*F*_(__1_, _72__)_ = 4.758; *p* = 0.032]. This also highlights greater overestimation among the ED group (*M* = 1.61, SD = 1.559) compared to the control group (*M* = 0.86, SD = 1.265).

**FIGURE 3:**
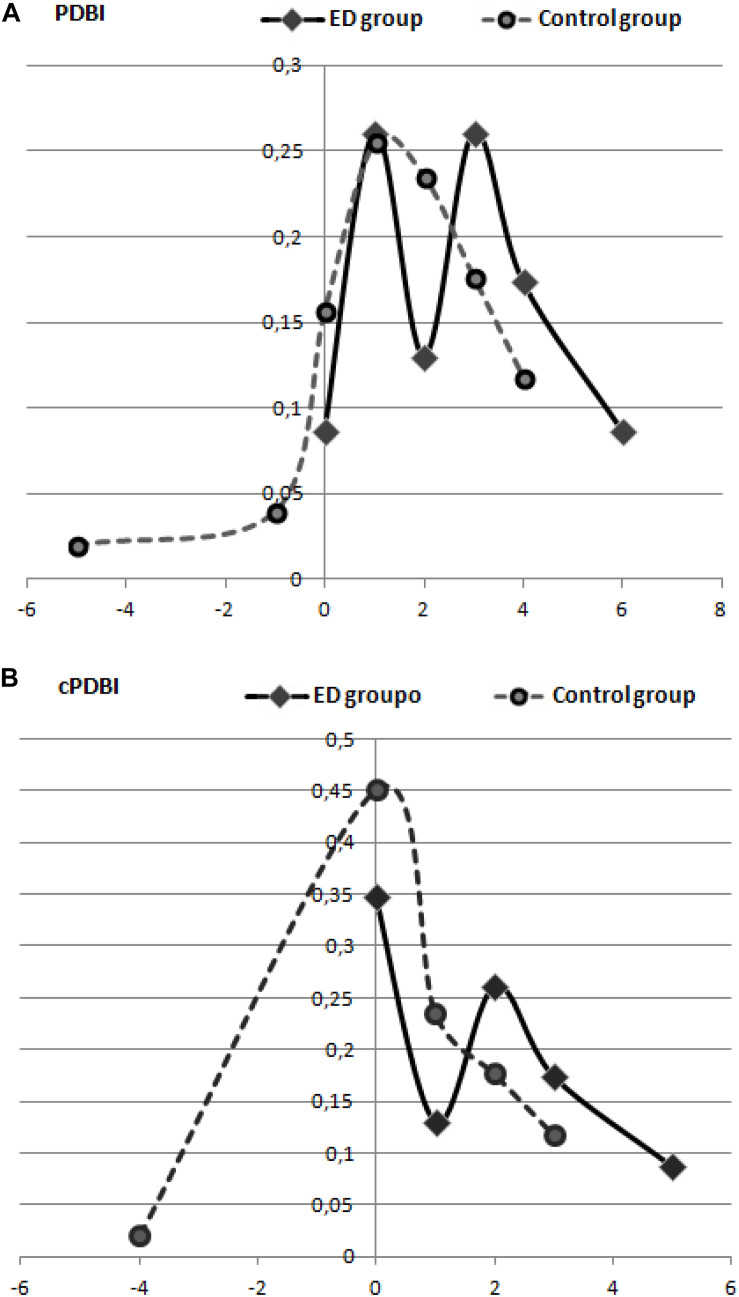
Percentage of representation of perceptual distortion of body image (PDBI) in the ED and control groups **(A)**, and adaptation using cPDBI **(B)**.

The results of PDBI showed statistically significant differences between groups (*p* = 0.028). The results of our modification, described in Section “Materials and Methods” (cPDBI), were also significant (*p* = 0.032). This PDBI estimated using the indices showed a 91.7% distortion of the body in the ED group compared to 84.3% in the control group (Figure 3A). The values of the cPDBI were 65.2 and 54.9% for the ED and control groups, respectively (Figure 3B).

### Associations IGT–PDBI: Hypothesis (d)

**FIGURE 4:**
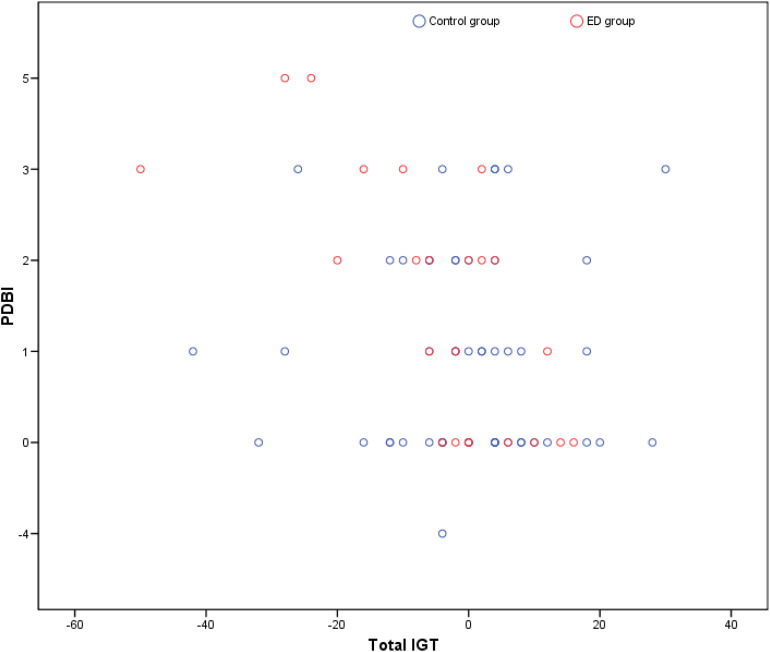
Scatter plot for the correlation between PDBI and total IGT score.

The correlations between the IGT and variables of body image distortion were analyzed. A negative correlation was obtained between the IGT and PDBI (*τ_*b*_* = −0.175; *p* = 0.045) and between the IGT and the cPDBI (*τ_*b*_* = −0.207; *p* = 0.021). Thus, the greater the distortion in body image, the smaller the IGT, which means that participants with more PDBI made riskier decisions (Figure 4). In the regression analysis, the independent variable “total IGT” explained 5.5% of the variance (R2) of the dependent variable “PDBI” (β = −0.234, 95% confidence interval, −0.056 to −0.001, *p* = 0.044) and it also explained 8% of the dependent variable “cPDBI” (β = 0.283, 95% confidence interval, −0.051 to 0.006, *p* = 0.014).

Furthermore, pBMI and cBMI showed a positive strong correlation (*τ_*b*_* = 0.399; *p* < 0.001) indicating that their self-body perception reflect the body reality. This correlation was stronger for the control group (*τ_*b*_* = 0.431; *p* < 0.001) than for the ED group (*τ_*b*_* = 0.381; *p* = 0.025), suggesting that control group perceived self-body images more accurately than subjects with ED. In the regression analysis, the independent variable “pBMI” explained 19.8% of the variance (R2) of the dependent variable “cBMI” (β = 0.457, 95% confidence interval, 0.225–0.604, *p* < 0.001).

## Discussion

The IGT is a useful test to assess the characteristics of everyday uncertainty in decisions and their consequences. Perception and cognitive and emotional DM processes might be affected in people suffering from ED. In this study, we hypothesized that the DM process is altered in young women with EDs.

The results supported our hypothesis and revealed significant differences between the ED and the control groups in the last two blocks of the computer-based version of the IGT. These differences were found in the last two blocks (after 60 trials), when members of the ED group tended to ignore long-term implications and made disadvantageous choices, making decisions contrary to those in previous trials in the first three blocks. This change in the direction of decisions produced economic losses. The control group tended to make more advantageous decisions, but they were still risky, as the total IGT for this group was also negative. This can be explained by the low mean age of both groups (teenagers), as well as the fact that all subjects were female.

The low mean age of both groups (teenagers) could be the explanation for this low total IGT, because as we mention in Introduction, IGT performance significantly improves from adolescence to adulthood (Hooper et al., 2004). Performance levels off after reaching the beginning of adulthood (approx. 19 years) (Overman and Pierce, 2013). Regarding sex, the study by van den Bos et al. (2013) indicated that women perform at lower levels than men. The authors proposed that women are hypersensitive to loss based on their greater preference for high-frequency payout cards (B and D decks) and the fact that they need 40–60 more trials than men to achieve the same performance level. Therefore, in our sample, both circumstances would explain the negative results in the total IGT: the young age (14.97 years) and that they would need up to 60 trials to improve performance.

In the results of the IGT per block, there were no significant differences between our groups in the first three blocks in accordance with previous research (Brand et al., 2007; Brogan et al., 2010; Chan et al., 2014), although slightly higher scores were found for the ED group. Another similarities with previous studies lies in the performance deficits obtained in the last two blocks of the IGT by ED patients. This suggests that members of the ED group learned the task better but showed worse execution; perhaps they could not or did not want inhibit the prepotent response of make a riskier choice. However, we did not find significant differences between the groups in terms of total IGT, as has been previously reported.

The mean age of participants in both groups in our study was 15 years. The mean age of participants in work by Brogan et al. (2010) was significantly different among the four groups and was higher than that of our sample. The mean ages reported in other studies were slightly different among groups, and were higher than our groups. Guillaume et al. (2010) tested participants with a mean age of 23 years for the ED group and 28 years for the control group. Chan et al. (2014) employed subjects with similar mean ages for the ED and control groups (∼26 years), as did Danner et al. (2012) (25 years). Consequently, the older ages of subjects in these studies and their heterogeneity between groups differ from ours, so they are not be comparable with regard to IGT execution. We consider the young age of our sample (approx. 15 years) and the similar age, educational and socioeconomic levels between groups as strengths of the present study. We understand that these similarities are key to establishing that possible differences in the IGT between groups should indeed be due to ED group main difference: PDBI.

As mentioned in the Introduction in the Age and Gender sections in relation to IGT performance, the age effect is useful for explaining discrepancies between the above-mentioned studies and our present work. They can be attributed to the maturation status of the PFC, specifically, structural changes and connection maturation after synaptic pruning occur in this brain region, which is involved in neurobiological decision-making processes (Overman and Pierce, 2013; van den Bos et al., 2013; Hartley and Somerville, 2015; Dumontheil, 2016). Active PFC development occurs throughout adolescence and is associated with seeking new experiences and engaging in risky behavior, which minimized the differences in terms of total IGT between our groups, but not in other studies with older subjects (Feinberg, 2017). However, our research on IGT performance during adolescence was not designed to determine the underlying neural bases for behavior changes. Rather we studied behavioral changes in IGT performance between two groups of adolescents: ED and control.

In terms of learning or evolution throughout blocks of the IGT, our results were in accordance with previous results reported by Brogan et al. (2010), who also found significant differences between ED patients and controls in the last two blocks of the test. Furthermore, Chan et al. (2014) observed differences in the third and fourth blocks (b3 and b4), with a tendency toward significant differences in the fifth block (b5). These consistencies, even with different mean ages among the studies, suggest that DM deficits in ED patients can be detected in adolescence, and support the concept proposed by Brogan et al. (2010) regarding ED progression. Thus, our findings significantly contribute to knowledge of the relationship between EDs and DM, with possible implications for preventing some characteristics of this disorder. In summary, in terms of total IGT, the small differences found between our adolescent groups would be exacerbated over time due to PFC maturation.

Consistent with Damasio’s theory (1994), the results of this study suggest that during the first 60 trials, the somatic markers of the consequences of each choice were created, and the subsequent 40 choices were guided by this past learning (psychophysiological memories of its consequences or secondary emotions) and fundamentally according to their objectives. Fuglset (2019) review indicates that the use of different imaging tests (e.g., embedded figures, fragmented images, and the Rey Complex Figures Test) suggests that patients with AN have weak central coherence or global integration difficulties, suggesting that these patients have greater local processing or a bias toward detail processing (Lang et al., 2014). It also refers to the few studies that have used the IGT to investigate decision making in AN already recovered (AN-REC). The results are contradictory since Tchanturia et al. (2007) did not find differences with the control group, while Danner et al. (2012) observed poorer decision-making than in controls. Therefore, it is still unknown whether ED neuropsychological deficits are related to predisposing traits or are a consequence of this disease. Thus, our results represent an advance in the knowledge of ED patient performance in relation to risk decision making and self-perception of their own body image. This might also explain the discrepant results between the groups in terms of partial IGT.

As Damasio (1994, 1996) hypothesize, somatic markers have positive or negative associations based on personal experience (hypothetical or imagined) of the consequences perceived as pleasant or aversive. The results suggest that for the ED group, risk decisions are somatically marked with pleasant emotions because they continue to take risks in the following trials despite their losses. This might be similar to the increase in adrenaline before vomiting, even if it has an initially unpleasant body sensation or momentary displeasure, which is surpassed by their pleasure to achieve their main objective: weight loss. As a result, they ignore the long-term negative consequences of their decisions (malnutrition/death). In the control group, risky decisions may be initially pleasant or attractive, as in the ED group (due to their similar ages, novel sensation seeking, and risk), but these subjects may consider long-term adverse consequences, which leads them to inhibit this attraction and make more advantageous choices.

## Conclusion

The inter-group differences obtained in the last two blocks task performance of this study suggest a kind of blindness toward the future in the ED group, as described by the developers of the SMH. Finally, the negative correlations found between the IGT and PDBI indicate that high distortions in body image are related to a lower gambling index, that is, these distortions are related to more disadvantageous or riskier decisions.

## Data Availability Statement

The raw data supporting the conclusions of this article will be made available by the authors, without undue reservation.

## Ethics Statement

The studies involving human participants were reviewed and approved by Ethics Committee for clinical trials of the “Juan Ramón Jiménez” Hospital of Huelva (Spain), PEIBA (Portal de Ética de la Investigación Biomédica de Andalucía). Written informed consent to participate in this study was provided by the participants’ legal guardian/next of kin.

## Author Contributions

All authors contributed substantially to the design and planning of the study, as well as in making decisions on the data provided by the legal guardians of the participants. They also contributed equally to the data analysis and interpretation and to writing and reviewing the manuscript. All authors approved the final version of the manuscript.

## Conflict of Interest

The authors declare that the research was conducted in the absence of any commercial or financial relationships that could be construed as a potential conflict of interest.
